# Reproducible induction of paroxysmal atrial fibrillation originating from the right atrial appendage by adenosine triphosphate

**DOI:** 10.1016/j.hrcr.2025.08.022

**Published:** 2025-08-25

**Authors:** Yasuteru Yamauchi, Tetsuya Asakawa, Yuichiro Sagawa, Kazuya Murata, Tetsuo Sasano, Kazutaka Aonuma

**Affiliations:** 1 Department of Cardiology, Japan Red Cross Yokohama City Bay Hospital, Kanagawa, Japan; 2 Department of Cardiology, Yamanashi Kosei Hospital, Yamanashi, Japan; 3 Department of Cardiovascular Medicine, Institute of Science Tokyo, Tokyo, Japan; 4 Department of Cardiology, Mito Saiseikai General Hospital, Ibaraki, Japan

**Keywords:** Atrial fibrillation, Non–pulmonary vein, Right atrial appendage, Adenosine triphosphate, Reproducible induction

**Key Teaching Points**

• Adenosine triphosphate (ATP) injection after pulmonary vein (PV) isolation may not only uncover dormant PV conduction but also reveal rare non-PV triggers*,* including those from the right atrial appendage (RAA).
• The reproducible induction of identical activation sequences by ATP indicates a strong association between ATP-induced non–pulmonary vein atrial fibrillation and clinically observed atrial fibrillation (AF).
• The RAA demonstrates distinct electrophysiological characteristics, including high-frequency, high-amplitude electrical signals and the shortest AF cycle length among all atrial regions, indicating heightened susceptibility to triggered activity.

## Introduction

Non–pulmonary vein (non-PV)–origin atrial fibrillation (AF) accounts for approximately 10%–30% of all AF cases. Isoproterenol (ISP) is commonly used to induce AF; however, in some cases of non-PV–origin AF, ISP cannot induce AF, while adenosine triphosphate (ATP) may sometimes be effective. ISP-induced AF is more likely to originate from the pulmonary vein (PV) (93%) compared with ATP-induced AF (50%), whereas ATP-induced non-PV triggers were found to predominantly originate from the right atrium (RA).^1^ The most common source of ATP-induced AF is the superior vena cava (SVC), whereas the right atrial appendage (RAA) accounts for only 0.3% of non-PV–origin AF cases. However, the clinical significance of ATP-induced AF currently remains unknown. Herein, we report a case of non-PV–origin AF naturally documented to originate from the RAA, with abnormal excitation reproducibly arising from this site with 100% consistency upon ATP injection. During AF, the excitation cycle length at the RAA was the shortest, and AF was terminated by focal ablation. Thereafter, AF could no longer be induced by ATP re-injection. This case demonstrates the utility of ATP in reproducibly triggering non-PV–origin AF.

## Case report

**Figure 1:**
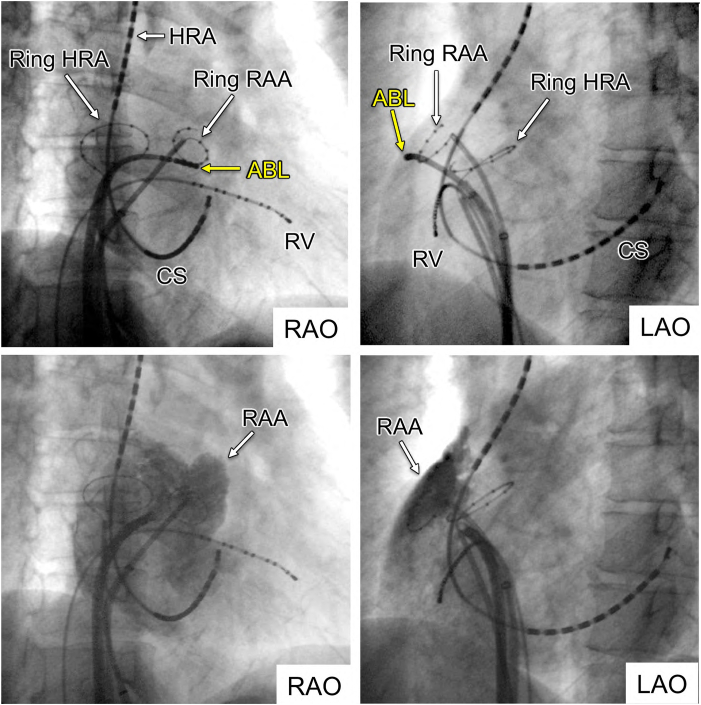
The successful ablation site and right atrial angiography. *Upper panel*: Small- and large-ring catheters were positioned in the RAA and HRA ostia, respectively. An ablation catheter was placed in the inferolateral RAA ostium. *Lower panel:* Right atrial angiography. The RAA is clearly visible. ABL = ablation catheter; CS = coronary sinus; HRA = high right atrium; LAO = left anterior oblique; RAA = right atrial appendage; RAO = right anterior oblique; Ring HRA = high right atrium; Ring RAA = right atrial appendage; RV = right ventricle.

**Figure 2:**
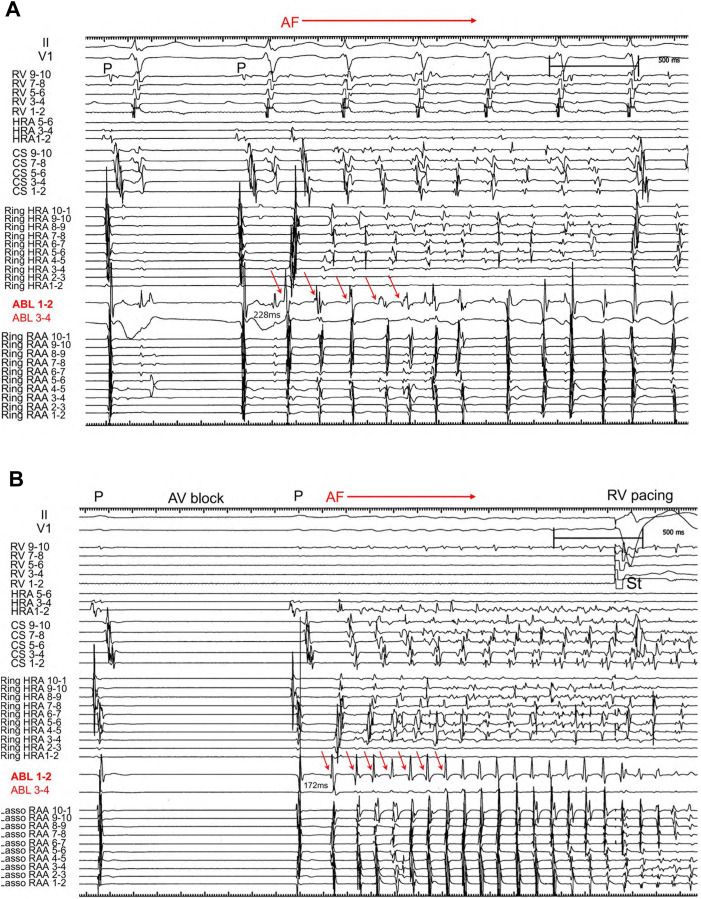
Intracardiac electrograms during spontaneous and ATP-induced AF. **A:** Intracardiac electrograms recorded during the initiation of spontaneous AF. The distal bipolar electrogram of the ablation catheter showed the earliest activation, with a coupling interval of 228 ms. **B:** Intracardiac electrograms during ATP-induced AF initiation showed that the earliest activation was recorded by the bipolar electrogram of the ablation catheter, with a coupling interval of 172 ms. The mean cycle length during 3 seconds of AF initiation was calculated as 138 ms for the CS catheter, 127 ms for the Ring HRA catheter, and 94 ms for the ablation and Ring RAA catheters. The *red arrows* indicated the non-PV trigger located at the earliest activation site. ABL = ablation catheter; AF = atrial fibrillation; ATP = adenosine triphosphate; AV = atrioventricular; CS = coronary sinus; HRA = high right atrium; P = P wave; RAA = right atrial appendage; Ring HRA = ring catheter positioned at the high right atrium; Ring RAA = ring catheter positioned at the right atrial appendage; RV = right ventricle.

**Figure 3:**
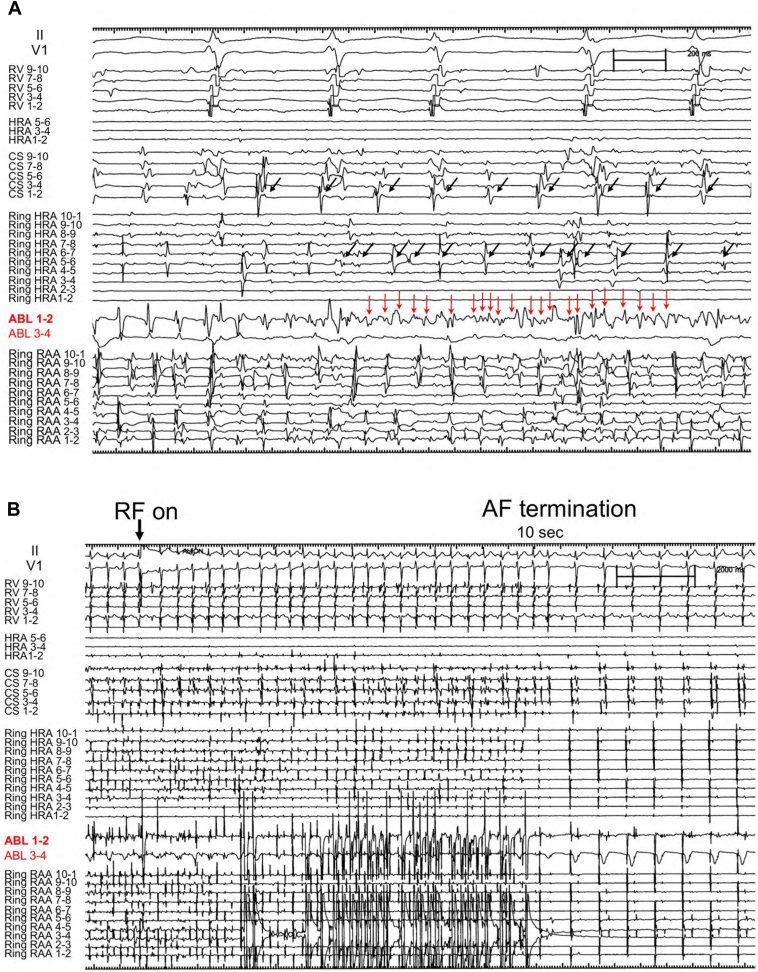
Intracardiac electrograms during AF and RF ablation. **A:** Intracardiac electrograms during AF revealed fractionated potentials (*red arrows*) at the ablation catheter positioned at the RAA base. The *black arrows* indicated electrograms recorded at the coronary sinus and high right atrium, where the cycle length was clearly longer than that at the RAA. **B:** AF was terminated 10 seconds after RF ablation at the RAA base. ABL = ablation catheter; AF = atrial fibrillation; CS = coronary sinus; HRA = high right atrium; RAA = right atrial appendage; RF = radiofrequency; Ring HRA = ring catheter positioned at the high right atrium; Ring RAA = ring catheter positioned at the right atrial appendage; RV = right ventricle.

**Figure 4:**
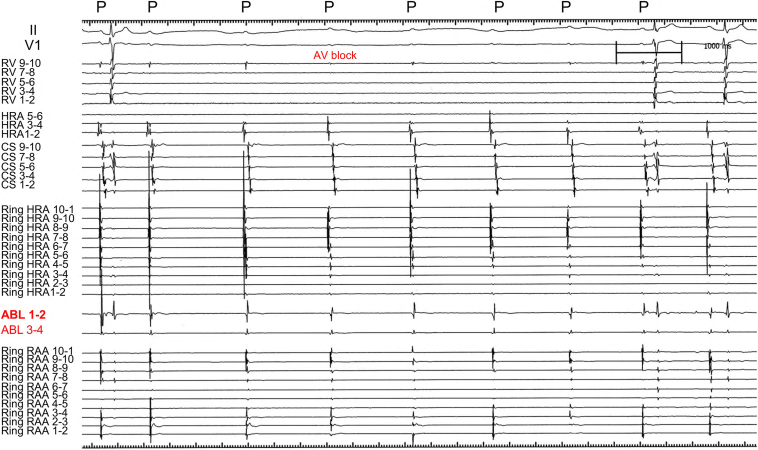
Intracardiac electrogram during ATP injection after focal ablation at the right atrial appendage. ATP injection–induced atrioventricular block. However, AF was not induced by ATP injection after RAA ablation. ABL = ablation catheter; AF = atrial fibrillation; ATP = adenosine triphosphate; AV = atrioventricular; CS = coronary sinus; HRA = high right atrium; P = P wave; RAA = right atrial appendage; Ring HRA = ring catheter positioned at the high right atrium; Ring RAA = ring catheter positioned at the right atrial appendage; RV = right ventricle.

A 35-year-old man was referred for catheter ablation of paroxysmal AF. Echocardiography revealed a normal ejection fraction and a left atrial diameter of 35 mm. First, we performed PV isolation, SVC isolation, and cavotricuspid isthmus ablation procedures using an 8-mm large-tip radiofrequency (RF) catheter (Ablaze Fantasista, Japan Lifeline). After ablation, 20 mg of ATP was injected to assess dormant PV conduction. While dormant PV and SVC conduction were not observed in any of the thoracic veins, ATP injections always induced non-PV–origin AF. Subsequently, non-PV trigger mapping was performed using 4-electrode catheters: 2 circular mapping catheters with diameters of 15 and 25 mm (Libero, Japan Lifeline), a 20-pole straight catheter (EPstar, Japan Lifeline), and a 20-pole diagnostic catheter positioned from the RA to the coronary sinus (CS) (BeeAT, Japan Lifeline). During the initiation of non-PV AF, electrograms of the CS catheter were always proximal to distal in direction, and electrograms of the high RA (HRA) region always preceded those of the CS. Therefore, by performing repeat ATP bolus injections, we attempted to comprehensively identify the earliest atrial activation site of non-PV trigger initiation originating around the HRA using 2 ring catheters of different sizes, a straight decapolar catheter, and an ablation catheter. Finally, the precise origin of the non-PV trigger was precisely localized to the base of the RAA (Figure 1, upper panel). The RA angiogram clearly showed the entire RAA, demonstrating that the small-ring catheter was positioned inside the RAA, while the tip of the ablation catheter was positioned at the base of the RAA more proximal to the small-ring catheter (Figure 1, lower panel). During spontaneous AF initiation, the distal pair bipolar electrogram of the ablation catheter showed the earliest atrial activation, with a coupling interval of 228 ms, preceding the electrogram of the small ring positioned into the RAA and the large ring positioned at the HRA (Figure 2A). Similarly, during ATP-induced AF initiation, the distal pair electrogram of the ablation catheter showed the earliest atrial activation, with a shorter coupling interval of 172 ms, compared with that during spontaneous AF initiation (Figure 2B). Furthermore, the mean cycle lengths during 3 seconds of AF initiation were 138, 127, and 94 ms for the CS, Ring HRA, and Ring RAA catheters, respectively, demonstrating that the RAA catheter had the shortest cycle length. The potentials of the CS and HRA catheters exhibited relatively long cycles, even during AF, whereas the potentials of the ablation catheter positioned at the base of the RAA during AF showed continuous or fractionated potentials (Figure 3A). We subsequently initiated RF ablation, with AF terminated 10 seconds after initiation. Subsequently, additional RF ablation was performed. After planar ablation to a certain extent at the base of the RAA, AF could no longer be induced, even with repeated ATP injections (Figure 4). The patient has remained free of AF for more than 10 years.

## Discussion

In recent years, ATP has emerged as a valuable pharmacological agent for unmasking latent non-PV triggers of AF after PV isolation.^1^^,^^2^ The mechanisms underlying ATP-induced AF involve activation of adenosine A1 receptors and G protein-coupled inwardly rectifying potassium channel subunit 4 channels, leading to membrane hyperpolarization and significant shortening of the action potential duration and effective refractory period, particularly in the RA, where these receptors and channels are more densely expressed.^3^ This shortening of atrial repolarization not only facilitates the initiation of triggered activity but may also contribute to the maintenance of AF by promoting a substrate conducive to reentry. This regional heterogeneity renders patients with RA more susceptible to ATP-induced AF.

Among the different RA structures, the RAA is rarely reported as a source of AF; however, recent studies and clinical observations suggest that its arrhythmogenic potential may be underrecognized. In a cohort of 317 consecutive ablation cases, RAA-origin AF was documented in approximately 7% of patients, predominantly male patients with paroxysmal AF but no structural heart disease.^4^ Moreover, the RAA exhibits unique electrophysiological properties, such as high-frequency, high-amplitude electrical signals and the shortest AF cycle length among all atrial regions (134.0 ± 10.9 ms), suggesting a predisposition to triggered activity.^5^

Tao et al^6^ previously demonstrated that ATP reproducibly induces AF from the RA, including the RAA, in a manner consistent with spontaneous AF triggers, thus supporting the clinical relevance of ATP-provoked arrhythmias. Our observations align with these findings. In the present case, we encountered reproducible ATP-induced AF originating from the RAA in the absence of PV reconnection. This phenomenon is rare but clinically important, particularly when mapping identifies the RAA as the earliest activation site. In contrast, in a study by Zhang et al,^2^ none of the 39 patients with ATP-induced AF post-PV isolation exhibited RAA-origin triggers, and the SVC was identified the predominant non-PV focus. This discrepancy may reflect the differences in patient selection and mapping resolution. In particular, prior studies may have underreported RAA-origin AF because of insufficient diagnostic catheter coverage, which could have limited the accurate delineation of the earliest activation sites. Our case underscores the importance of comprehensive spatial sampling using multipolar mapping catheters for precise localization of non-PV triggers in the RA. Nonetheless, the consistent ATP inducibility of AF from the RAA observed in other reports, along with our own experience, underscores the need to consider the RAA as a possible arrhythmogenic source for managing recurrent AF. This consideration is particularly important in younger patients, who warrant a higher index of suspicion for potential non-PV triggers, including other forms of supraventricular tachycardia.

The unique anatomical and physiological characteristics of the RAA, including its trabeculated structure, variable autonomic innervation, and potential embryological remnants, may contribute to its vulnerability to ATP-induced activity. Additionally, ATP-induced transient bradycardia may potentially create a favorable milieu for abnormal automaticity and early afterdepolarization, further enhancing the arrhythmogenicity in this region. In the present case, the trigger site for ATP-induced AF was identical to that for spontaneous AF. This represents a valuable example, suggesting that the trigger is likely the same as that responsible for clinical AF when AF is reproducibly induced by ATP from the same site.

## Conclusion

Overall, this case demonstrates that the RAA can serve as an ATP-induced non-PV trigger in patients with AF. Precise mapping and focal ablation of the RAA successfully eliminated arrhythmia, thus highlighting the utility of ATP testing for uncovering rare arrhythmogenic sites. The reproducible induction of identical activation sequences by ATP suggests a strong correlation between ATP-induced non-PV AF and clinical AF, underscoring the clinical significance of these arrhythmias.

## Disclosures

The authors have no conflicts of interest to disclose.

## References

[1] Tutuianu C., Pap R., Riesz T., Bencsik G., Makai A., Saghy L. (2019). Is adenosine useful for the identification of atrial fibrillation triggers?. J Cardiovasc Electrophysiol.

[2] Zhang J., Tang C., Zhang Y., Su X. (2014). Origin and ablation of adenosine triphosphate-induced atrial fibrillation after circumferential pulmonary vein isolation: effects on procedural success rate. J Cardiovasc Electrophysiol.

[3] Li N., Csepe T.A., Hansen B.J. (2016). Adenosine-induced atrial fibrillation: localized re-entrant drivers in the lateral right atrium due to the heterogeneous expression of adenosine A1 receptors and GIRK4 subunits in the human heart. Circulation.

[4] Baptiste F., Kalifa J., Durand C. (2022). Characteristics and prevalence of right atrial appendage-originating AF. Front Cardiovasc Med.

[5] Liu Y., Song Z., Jiang W., Wu S., Liu X., Qin M. (2022). Right atrial appendage: an important structure to drive atrial fibrillation. J Interv Card Electrophysiol.

[6] Tao S., Yamauchi Y., Maeda S. (2014). Adenosine triphosphate-induced atrial fibrillation: clinical significance and relevance for spontaneous atrial fibrillation. J Interv Card Electrophysiol.

